# Clinical pharmacist-led problem-specific education as a strategy for addressing suboptimal antimicrobial use in intensive care unit: a prospective pre-post analysis

**DOI:** 10.3389/fphar.2025.1556884

**Published:** 2025-05-26

**Authors:** Enes Emir İlerler, Yunus Emre Ayhan, Erdem Yalçinkaya, Sait Karakurt, Mesut Sancar

**Affiliations:** ^1^ Department of Clinical Pharmacy, Marmara University, Istanbul, Türkiye; ^2^ Department of Clinical Pharmacy, Prof. Dr. Cemil Taşcıoğlu City Hospital, Istanbul, Türkiye; ^3^ Department of Pulmonary and Critical Care Medicine, Marmara University, Istanbul, Türkiye

**Keywords:** antimicrobial drug therapy problems, intensive care unit, clinical pharmacist-led services, suboptimal antimicrobial use, clinical pharmacists, antimicrobial stewardship

## Abstract

**Background:**

Antimicrobial use in ICUs is challenging due to altered pharmacokinetics, severe infections, and the burden of comorbidities. This study aims to investigate the contribution of clinical pharmacy services in reducing antimicrobial drug therapy problems (ADTPs) in the intensive care unit.

**Methods:**

This study was a prospective, pre-post intervention study conducted over a total duration of 6 months (15 January 2023–15 July 2023) in Türkiye. During both control period (CP) and intervention period (IP), ADTPs were identified and classified according to established definitions describing each day of therapy with a specific antimicrobial agent. In IP, clinical pharmacist-led services were implemented for the ICU team, encompassing problem-targeted educational sessions and bedside intervention recommendations.

**Results:**

A total of 85 patients (CP, n = 43; IP, n = 42) were included in the study. The mean age of the patients was 68.87 years (SD = 16.09). The most common indication for antimicrobial initiation was pneumonia (56.5%), while the most frequently used antimicrobial agent throughout the study was piperacillin-tazobactam (44.7%). It was found that 5.5% of patients across all periods received unnecessary, 2.2% inappropriate, and 92.3% sub-optimal antimicrobial therapy. During both CP and IP, almost all ATDPs were categorized under sub-optimal treatment problems related to medication dosage and/or administration regimens (93.94% vs. 88%). A statistically significant 62% reduction in total ADTPs was observed during IP compared to CP (total ADTPs, 66 vs. 25; p = 0.001).

**Conclusion:**

This study identified a high incidence of ADTPs in the ICU, with the majority classified as sub-optimal. The significant reduction in ADTPs observed between the periods with the provision of clinical pharmacy services highlights the effective role of clinical pharmacists in reducing ADTPs.

## 1 Introduction

Intensive care units (ICUs) present an elevated risk for infections due to the critical condition of patients, the presence of multiple comorbidities, high-risk surgeries, and the frequent use of invasive devices, such as central venous catheters and endotracheal tubes. According to the 2019 epidemiological surveillance report by the European Centre for Disease Prevention and Control, 8,874 ICU patients (7.4%) who stayed for more than 2 days developed at least one ICU-acquired healthcare-associated infection (HAI), including pneumonia, bloodstream infections (BSIs), and urinary tract infections (UTIs) (Pons and Ruiz, 2019; European Centre for Disease Prevention and Control, 2019).

Inappropriate or inadequate antibiotic therapy for bacterial infections in the ICU significantly increases mortality risk, while unnecessary antibiotic use not only heightens the risk of adverse drug reactions and complications like *Clostridium difficile* infections but also exacerbates the global crisis of antimicrobial resistance (AMR), a leading public health threat associated with an estimated 10 million annual deaths by 2050 if unmitigated (O’Neill, 2016; World Health Organization, 2024). Approximately 20%–50% of hospitalized patients, and 30%–60% of ICU patients, are prescribed unnecessary, inappropriate, or suboptimal antibiotic therapy (Kumar et al., 2009; Kumar et al., 2006). Variations in drug clearance due to altered pharmacokinetics in critically ill patients, particularly those with sepsis, can lead to subtherapeutic antibiotic concentrations with standard dosing regimens. Early and appropriate antimicrobial therapy remains essential for improving outcomes in critically ill patients (Luyt et al., 2014; Marquet et al., 2015; Schuts et al., 2016; Guclu et al., 2017; Heffernan et al., 2021).

The integration of a clinical pharmacist into the multidisciplinary ICU team is essential to address these challenges. Clinical pharmacists provide expertise in detecting prescribing errors, adjusting dosages, and monitoring for polypharmacy-related adverse effects, thereby optimizing antibiotic therapy. This approach has been linked to improved outcomes, including reduced ICU length of stay and mortality, along with cost benefits (Leape et al., 1999; Stollings et al., 2018; Montazeri and Cook, 1994). Clinical pharmacists also play a vital role in educating the ICU team on evidence-based pharmacotherapy and facilitating targeted drug strategies for patients with multiple organ failure, ensuring a more individualized and effective treatment approach (McKenzie et al., 2024).

While clinical pharmacists have emerged as pivotal contributors to antimicrobial stewardship (AMS) through therapeutic monitoring and guideline implementation, transient knowledge gaps caused by frequent physician rotations in ICUs often undermine adherence to evidence-based practices. This is the first study in Türkiye to evaluate clinical pharmacist-led services for antimicrobial optimization in ICUs. Furthermore, this is the first study to apply Spivak et al.’s classification system (Spivak et al., 2016) in an ICU population, offering a novel methodology for categorizing ADTPs in critically ill patients. The study focuses on high-risk ICU subpopulations, including: Elderly patients with high comorbidity burdens), Patients receiving invasive mechanical ventilation (IMV), who are at heightened risk for ventilator-associated pneumonia (VAP) and prolonged antibiotic exposure, critically ill patients with sepsis/septic shock, a population requiring rapid, precise dosing adjustments due to altered pharmacokinetics. While previous Turkish studies have addressed antibiotic consumption patterns (Guclu et al., 2017), none have assessed the impact of pharmacist-driven interventions on ADTP classification and reduction.

This study introduces a novel clinical pharmacist-led strategy combining structured, problem-targeted educational sessions with real-time bedside interventions to address ADTPs. Unlike prior interventions focusing solely on education or retrospective audits, this approach directly integrates pharmacist expertise into daily ICU workflows, addressing both systemic knowledge gaps and patient-specific therapeutic challenges.

This study aims to identify and classify antimicrobial drug therapy problems (ADTPs) and evaluate the impact of clinical pharmacy services on reducing these problems in the ICU setting. Using a pre-post intervention design, we directly compare ADTP rates before and after implementing clinical pharmacist-led services. This approach isolates the intervention’s effect while controlling for baseline institutional practices and patient characteristics, thereby strengthening the validity of our findings by minimizing confounding temporal trends. The study provides a robust framework to assess the effectiveness of targeted educational and bedside interventions in optimizing antimicrobial use.

## 2 Materials and methods

### 2.1 Study design and setting

This prospective, pre-post intervention study was conducted in the Medical Intensive Care Unit of a tertiary education and research hospital (13-bed) in Türkiye, from 15 January 2023, to 15 July 2023. Adult patients hospitalized for at least 24 h and who received at least one antimicrobial treatment, either upon admission or during their hospital stay, were included in the study. Patients admitted to the intensive care unit on weekends and those who could not be followed up were excluded from the study. Patients were assigned to the study without randomization during the study periods.

The study was designed to include two distinct periods, each lasting approximately 3 months. Throughout the study period, the clinical pharmacy conducted patient evaluations and participated in routine patient rounds on weekdays. The first follow-up period was the control period, during which patients were monitored by the clinical pharmacy without receiving any recommendations regarding the appropriateness of their antimicrobial therapy (dose, antimicrobial selection, duration of treatment, or route of administration). Any identified ADTPs were recorded. The second follow-up period was the intervention period. Unlike the observation period, clinical pharmacist-led services were provided to ICU team. The clinical pharmacist participated in routine patient rounds 5 days a week.

### 2.2 Data collection

Patient data, including sociodemographic characteristics, medical and medication history, daily treatment orders, routine laboratory and biochemical parameters, culture results, and clinical scores, were extracted from the Electronic Health Records (ORIGO Hospital Information Management System). Specific variables collected comprised Glasgow Coma Scale (GCS) and Sequential Organ Failure Assessment (SOFA) scores, Charlson Comorbidity Index (CCI), ICU admission reasons, antimicrobial therapy initiation rationale, mechanical ventilation and renal replacement therapy status, vasopressor use and another important variables. These data were selected based on their relevance to antimicrobial therapy management in critically ill patients.

### 2.3 Identification and classification of antimicrobial drug treatment problems

Definitions of terminology to describe a day of therapy for particular antimicrobial agent suggested by Spivak et al., which categorizes antimicrobial therapies as “unnecessary,” “inappropriate,” or “sub-optimal,” was used to identify and classify ADTPs during both the control and intervention periods of the study (Spivak et al., 2016). According to the established terminology, an ADTP classified as “unnecessary” meets at least one criterion indicating use of antimicrobials without clinical justification, such as administration in non-infectious cases, treatment of nonbacterial infections, continuation beyond the indicated duration without need, redundant therapy, or continued broad-spectrum use after pathogen identification. An ADTP classified as “inappropriate” meets at least one criterion indicating “use of antimicrobials in cases where the causative pathogen is resistant” or use that deviates from established treatment guidelines. An ADTP classified as “sub-optimal” meets at least one criterion indicating use of antimicrobials in a confirmed infection where therapy could be improved, specifically by adjusting the drug choice, administration route, or dosage.

During both periods, the clinical pharmacist evaluated the antimicrobial treatment in terms of efficacy, dosing, treatment duration, adherence to national and international guidelines, dilution methods, routes of administration of drugs, drug selection or discontinuation, and possible drug-drug interactions at the “Major” (D category) and “Contraindicated” (X category) levels. UpToDate^®^ Drug Information (Wolters Kluwer, 2023), Micromedex^®^ Drug Information (IBM Watson Health, 2023), Sanford Guide to Antimicrobial Therapy (Gilbert et al., 2023), and national and international guidelines (Turkish Thoracic Society Community-Acquired Pneumonia Guideline (Özlü et al., 2009), Management of Adults with Hospital-acquired and Ventilator-associated Pneumonia (Erb et al., 2016), Surviving Sepsis Campaign Guidelines (Evans et al., 2021a)) were used to evaluate ADTPs and provide appropriate intervention recommendations. Any identified problems were recorded after reaching a consensus between the clinical pharmacist and the responsible Intensive Care Specialist. To ensure consistency, ADTP classifications were guided by predefined criteria from Spivak et al. (2016) and disagreements were resolved through iterative discussions until unanimous agreement was achieved.

The consumption frequencies of the antibiotics studied were analyzed based on the World Health Organization’s (WHO) AWaRe classification, which categorizes antibiotics into Access, Watch, and Reserve groups (World Health Organization, 2019). This classification system was designed to enhance antibiotic stewardship efforts by facilitating the monitoring of antibiotic usage patterns, with a specific focus on reducing antimicrobial resistance at regional and national levels. Antibiotics in the “Access” group are effective against commonly encountered pathogens and present a lower risk for resistance development compared to other groups. The “Watch” group includes antibiotics with a relatively higher risk of promoting bacterial resistance and are thus prioritized group for monitoring and management due to their critical role in human health. Finally, the “Reserve” group comprises antibiotics intended solely for use in treating infections confirmed or suspected to involve multidrug-resistant pathogens.

### 2.4 Clinical pharmacist-led services

**FIGURE 1:**
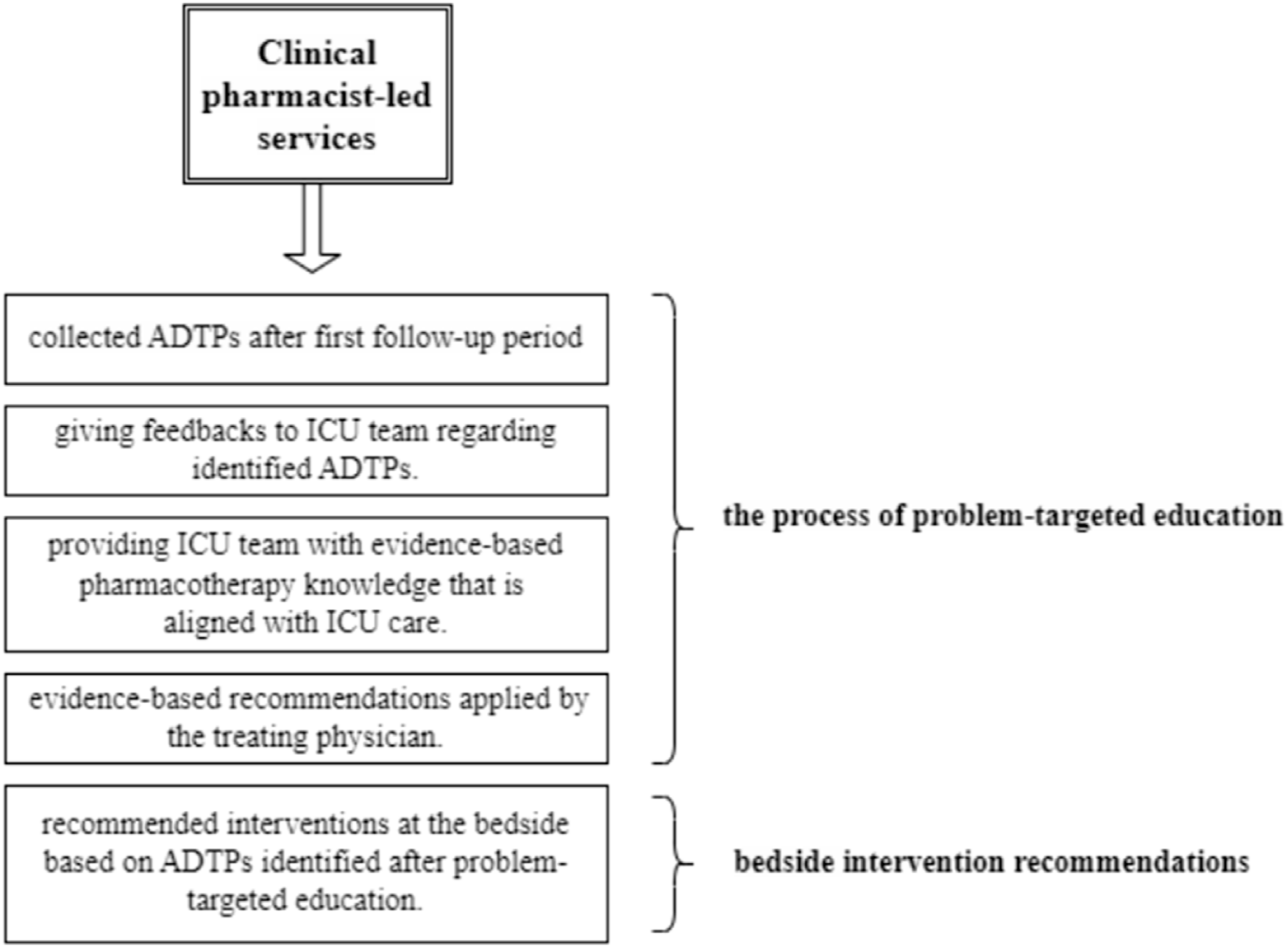
Workflow of clinical pharmacist-led services.

Due to the monthly rotation of physicians caring for patients in our intensive care unit, the problem-targeted education sessions were organized through face-to-face presentations for the ICU team during the first week of each month starting from the first month of intervention period. These sessions aimed to provide feedback on ATDPs identified during the observation period, to raise awareness about the potential negative consequences of inadequate/improper antimicrobial use and to promote optimal antimicrobial use through feedbacks and suggested evidence-based pharmacotherapy for ADTPs. Additionally, ADTPs identified during the intervention (after problem-targeted education sessions) were addressed through bedside intervention recommendations made by the clinical pharmacist (Figure 1). During the intervention period, ADTPs identified after education sessions were addressed through bedside recommendations. Adherence to these recommendations was tracked, with interventions documented as “accepted” or “rejected” in patient records. The ICU team’s acceptance rate was to reflect heightened awareness from evidence-based education, which emphasized guideline-aligned practices and the clinical consequences of suboptimal antimicrobial use.

### 2.5 Primary and secondary outcome measures

The primary outcome of the study is the reduction in ADTPs through clinical pharmacist-led interventions. The secondary outcomes of the study are to classify ADTPs, report their frequency of occurrence, and determine the frequency of antimicrobial agents associated with ADTPs in the ICU.

### 2.6 Sample size calculation

To calculate the sample size, data from Thomnoi et al. (2022), which reported a 22% improvement in dose appropriateness (from 78% to 100%) in groups receiving clinical pharmacist recommendations, were used (alpha 0.05, power 90%). Based on this calculation, at least 41 patients were required in each group. Considering a 15% loss to follow-up rate, it was decided to include a total of 94 patients in the study. The sample size calculation was performed using G-Power version 3.1.9.7.

### 2.7 Data analysis

The statistical analysis of the results was conducted using IBM SPSS statistics software version 25.0. Chi-square analysis was applied to compare categorical data, and the Kolmogorov-Smirnov/Shapiro-Wilk test was used to assess the normality of the distribution for continuous variables. Continuous variables with normal distribution were analyzed using the Student’s t-test, while non-normally distributed data were analyzed using the Mann-Whitney U test. Categorical variables were compared using the chi-square test or Fisher’s exact test, as appropriate. A p-value of <0.05 was considered statistically significant at the 95% confidence interval.

## 3 Results

### 3.1 Patient characteristics

**FIGURE 2:**
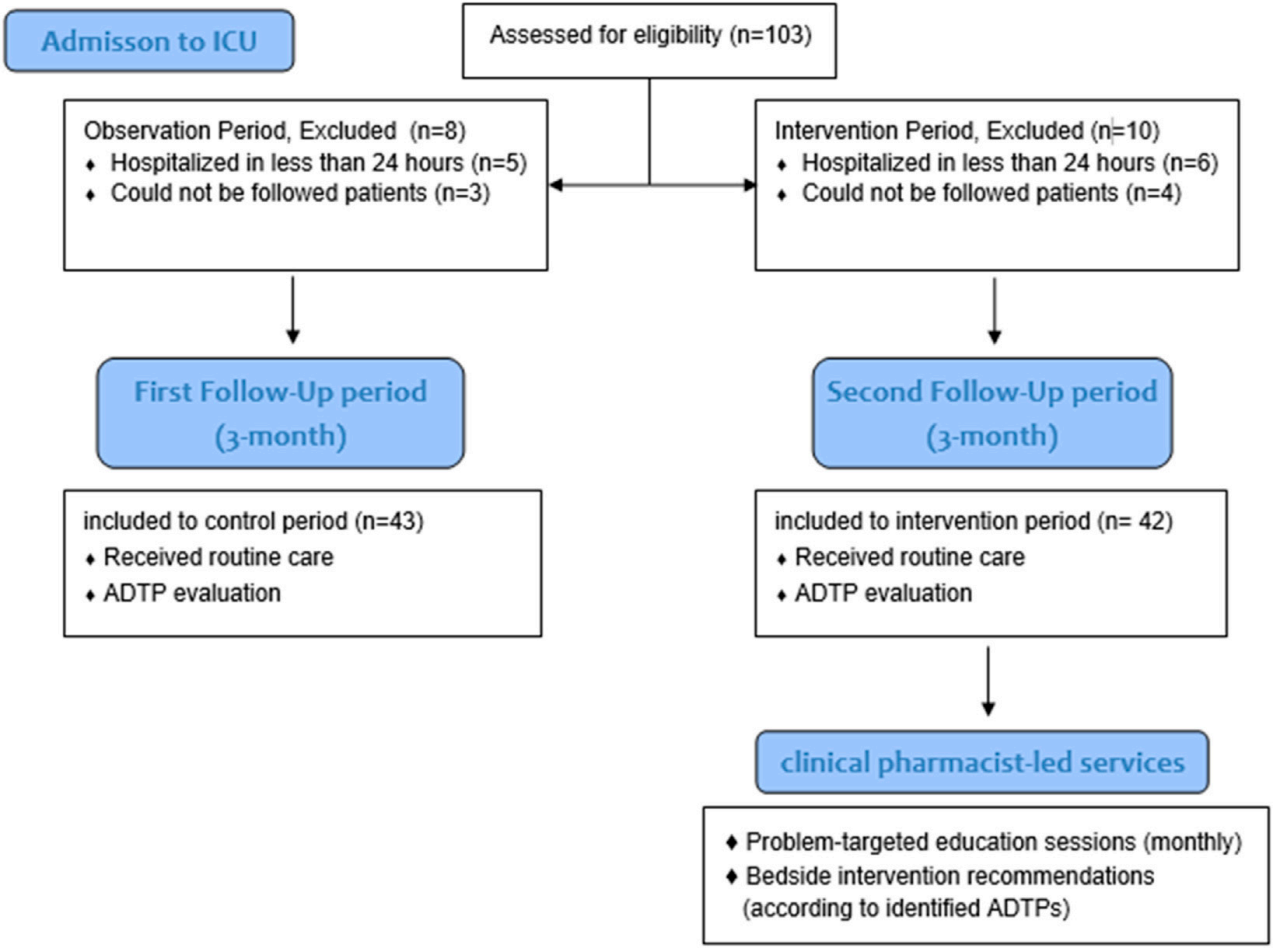
Participant enrollment and study phases.

A total of 103 patients were admitted to the ICU, with 85 included in the final analysis after excluding those with a hospital stay of less than 24 h or admitted over the weekend (Figure 2). The mean age of patients was 68.87 ± 16.09 years, and 50.6% were male (Table 1). Respiratory conditions, primarily pneumonia (47.1%), and circulatory conditions, primarily sepsis/septic shock (31.8%), were the most common reasons for ICU admission. The most frequent comorbidities were hypertension (43.5%) and oncological malignancies (30.6%) (Table 2). Significant differences were observed between the groups for age (mean, 72.79 vs. 64.86, p = 0.022), CCI (mean, 6.33 vs. 5.05, p = 0.021), GCS (median, 12 vs. 15, p = 0.037), and IMV (67.4% vs. 42.9%, p = 0.023).

**TABLE 1:** Demographic characteristics of patients.

Variable	Total (n = 85)	CP (n = 43)	IP (n = 42)	p-value
Age in years, mean (SD)	68.87 (16.09)	72.79 (13.15)	64.86 (17.9)	0.022
Gender, n (%)				0.447
Male	42 (49.4)	23 (53.5)	19 (45.2)	
Female	43 (50.6)	20 (46.5)	23 (54.8)	
CCI score, mean (SD)	5.69 (2.58)	6.33 (2.56)	5.05 (2.46)	0.021
SOFA score, mean (SD)	6.98 (4.34)	7.82 (4.90)	6.12 (3.55)	0.071
APACHE II score, mean (SD)	20.82 (7.46)	21.44 (8.38)	20.20 (6.42)	0.441
Number of medications on admission, mean (SD)	9.91 (3.38)	9.37 (3.62)	10.45 (3.05)	0.141
Number of comorbidities, median (IQR)	3 (1–4)	3 (1–4)	2 (1–4)	0.253
GCS score, median (IQR)	14 (7–15)	12 (3–15)	15 (11.75–15)	0.037
Total length of stay, median day (IQR)	7 (4–12)	7 (4–12)	7 (4.75–12.25)	0.540
Number of antimicrobial drugs per patient, median (IQR)	2 (2–3.5)	2 (1–3)	2.5 (2–4)	0.492

CCI, charlson comorbidity index; GCS, glasgow coma scale; CP, control period; IP, intervention period.

**TABLE 2:** Clinical characteristics of patients.

Variable	Total (n = 85)	CP (n = 43)	IP (n = 42)	p-value
Reason for admission, n (%)				0.356
Respiratory-related	40 (47.1)	20 (46.5)	20 (47.6)	
Circulatory-related	27 (31.8)	12 (27.9)	15 (35.7)	
Others	18 (21.2)	11 (25.6)	7 (16.7)	
IMV support, n (%)	47 (55.3)	29 (67.4)	18 (42.9)	0.023
IMV duration, median day (IQR)	4 (2–11)	3 (1–11.5)	4.5 (3–9)	0.234
Vasopressor use, n (%)	57 (67.1)	31 (72.1)	26 (61.9)	0.442
AKI, n (%)	45 (52.9)	26 (60.5)	19 (45.2)	0.160
CVVHD, n (%)	11 (12.9)	8 (18.6)	3 (7.1)	0.115
Sepsis/Septic Shock, n (%)	50 (58.8)	28 (65.1)	22 (52.4)	0.233
Reason for initiation of treatment, n (%)				0.161
Pneumonia	48 (56.5)	25 (58.1)	23 (54.8)	
Sepsis/Septic Shock	16 (18.8)	6 (14)	10 (23.8)	
Catheter-Related Bloodstream Infection	5 (5.9)	1 (2.3)	4 (9.5)	
Urinary Tract Infection	5 (5.9)	2 (4.7)	3 (7.1)	
Intra-abdominal Infection	8 (9.4)	7 (16.3)	1 (2.4)	
Others	3 (3.5)	2 (4.7)	1 (2.4)	
Culture growth, n (%)				0.919
positive	45 (52.9)	23 (53.5)	22 (52.4)	
negative	40 (47.1)	20 (46.5)	20 (47.6)	
Discharge Status, n (%)				0.063
Transferred to ward	46 (54.1)	19 (44.2)	27 (64.3)	
Death	39 (45.9)	24 (55.8)	15 (35.7)	

AKI, acute kidney injury; CVVHD, continuous venovenous haemodialysis; CP, control period; IP, intervention period; IMV, invasive mechanical ventilation; IQR, interquartile range.

Upon analysis, it was found that the most frequent indication for initiating antimicrobial therapy was pneumonia, accounting for 56.5% of cases, followed by sepsis or septic shock, which occurred in 18.8% of patients. Among the patients receiving antimicrobials, 52.9% exhibited positive culture growth. Additionally, 48.2% of patients were treated with targeted therapy. The distribution of these characteristics was comparable between the control and intervention groups.

Table 3 details the frequency of antimicrobial drug use in the intensive care unit (ICU). The most commonly prescribed agents were beta-lactam antibiotics, with piperacillin-tazobactam (44.7%) and meropenem (43.5%) being the predominant choices. Vancomycin was the next most frequently used agent, administered to 31.8% of patients. Among antifungal agents, anidulafungin and fluconazole were the primary drugs utilized in the ICU. Additionally, when comparing the frequency of drug use between the control and intervention periods, no statistically significant differences were observed.

**TABLE 3:** Frequency of antimicrobial agents used, stratified by WHO AWaRe classification.

AWaRe classification	Antimicrobials	Overall frequency (%)	CP, n (%)	IP, n (%)	p-value
Watch	Piperacillin-tazobactam	44.7	20 (46.5)	18 (42.9)	0.904
Watch	Meropenem	43.5	15 (34.9)	22 (52.4)	0.159
Watch	Vancomycin	31.8	14 (32.5)	13 (31.0)	1.000
Watch	Cefepime	21.2	9 (20.9)	9 (21.4)	1.000
Watch	Ciprofloxacin	15.3	8 (18.6)	5 (11.9)	0.578
Access	Ampicillin-sulbactam	11.8	5 (11.6)	5 (11.9)	1.000
Reserve	Polymyxins	10.6	3 (7.0)	6 (14.3)	0.458
Watch	Levofloxacin	9.4	4 (9.3)	4 (9.5)	1.000
Watch	Clarithromycin	9.4	2 (4.7)	6 (14.3)	0.250
Watch	Ceftriaxone	7.1	2 (4.7)	4 (9.5)	0.650
Watch	Imipenem	5.9	3 (7.0)	2 (4.8)	1.000
Access	Trimethoprim-sulfamethoxazole	5.9	1 (2.3)	4 (9.5)	0.343
Access	Cefazolin	4.7	2 (4.7)	2 (4.8)	1.000
Reserve	Daptomycin	4.7	3 (7.0)	1 (2.4)	0.625
Access	Amikacin	3.5	1 (2.3)	2 (4.8)	0.983
	Antifungals				
	Anidulafungin	5.9	3 (7.0)	2 (4.8)	1.000
	Fluconazole	4.7	3 (7.0)	1 (2.4)	0.625
	Others	12.9	6 (14.0)	5 (11.9)	1.000

CP, control period; IP, intervention period.

The most frequently isolated pathogen during the study was *Pseudomonas aeruginosa* (15.5%), followed by *Escherichia coli* (13.8%), *Enterococcus faecium* (13.8%), and *Klebsiella pneumoniae* (10.3%). MRSA and *Stenotrophomonas maltophilia* were found only during the control period, while *Candida auris* was detected exclusively during the intervention period.

A total of 91 ADTPs were identified, with unnecessary, inappropriate, and suboptimal therapies accounting for 5.5% (n = 5), 2.2% (n = 2), and 92.3% (n = 84) of the total ADTPs, respectively. ADTPs were detected in 90.7% of patients during the control period and 52.4% during the intervention, with suboptimal ADTPs being the most common in both periods (ADTPs 62 vs. 22; *t* = 4,19 p = 0.001). A significant 62% reduction in total ADTPs was observed during the intervention compared to the control period (ADTPs, 66 vs. 25; *t* = 4,66, p = 0.001) (Table 4).

**TABLE 4:** Classification of antimicrobial drug treatment problems.

ADTP category	CP (n = 43)			IP (n = 42)			p-value
	Number of ADTPs (%)	ADTPs per patient	Number of patients (%)	Number of ADTPs (%)	ADTPs per patient	Number of patients (%)	
Unnecessary	2 (3.03)	0.05	2 (4.7)	3 (12.00)	0.07	2 (4.8)	NA*
Inappropriate	2 (3.03)	0.05	2 (4.7)	—	—	—	NA*
Sub-optimal	62 (93.94)	1.44	35 (81.4)	22 (88.00)	0.52	20 (40.6)	0.001
Total ADTPs	66 (100)	1.53	39 (90.7)	25 (100)	0.59	22 (52.4)	0.001

ADTP, antimicrobial drug therapy problems; CP, control period; IP, intervention period.

* Not applicable.

### 3.2 Impact of specific interventions

Following problem-targeted education sessions, the total ADTPs decreased from 66 (control period) to 25 (intervention period), reflecting a 62% reduction attributable to education. Bedside intervention recommendations were then applied to these 25 ADTPs, with an 80% acceptance rate by the ICU team, reflecting behavioral changes driven by increased awareness of ADTP consequence This led to the resolution of 20 ADTPs (80% of 25), leaving 5 unresolved. The most impactful bedside interventions included optimizing beta-lactam dosing via prolonged infusions (addressing 40% of sub-optimal ADTPs). Education sessions primarily targeted sub-optimal dosing and administration problems. (i.e., beta-lactam premature dose reductions during acute kidney injury, optimizing beta-lactam dosing via prolonged infusions and inadequate dosing in patients receiving continuous renal replacement therapy (CRRT) (Table 5).

**TABLE 5:** Most common ADTPs Under Sub-optimal Category.

Individual ADTPs	CP		IP(Post-education)		
	ADTPs (n)	Percentage (%)^a^	ADTPs (n)	Percentage (%)^b^	Resolved via bedside interventions (n)
Failure to use prolonged infusion regimens to optimize the PK/PD targets of beta-lactams	33	50	10	40	10
Dose reduction of beta-lactams during the initial phase of sepsis-associated acute kidney injury (AKI) (within the first 24–48 h)	8	12.1	0	0	-
Lack of loading dose administration at the start of vancomycin therapy in critically ill/septic patients	7	10.6	0	0	-
Inadequate antimicrobial dosing in patients receiving continuous renal replacement therapy (CRRT)	6	9.1	0	0	-

CP, control period; IP, intervention period.

^a^ Values represent the proportion within the total of 66 ADTPs.

^b^ Values represent the proportion within the total of 25 ADTPs.

### 3.3 Subgroup and sensitivity analyses of ADTPs by age, comorbidity, and mechanical ventilation status

To evaluate the robustness of the study’s findings and address potential confounding variables (e.g., age, Charlson Comorbidity Index [CCI], invasive mechanical ventilation [IMV]), sensitivity analyses were conducted. The intervention reduced ADTPs significantly in both Geriatric (≥65 years, Δ = 60%, *U = 180*, p = 0.004) and Non-geriatric patients subgroups (<65 years Δ = 65%, *U = 90*, p = 0.002). Greater reductions were observed in the high-comorbidity group (CCI >5, Δ = 68%, *U = 140,* p = 0.001) compared to the low-comorbidity group (CCI ≤5, Δ = 54%*, U = 110* p = 0.012). ADTP reductions were significant in both IMV (Δ = 65%, U = 150 p = 0.001) and non-IMV (Δ = 58%, *U = 160*, p = 0.004) subgroups. In correlation analyses, CCI was weakly correlated with ADTPs (*r = 0.275*, p = 0.011), while no significant relationship was found between other variables and ADTPs. To test whether results were driven by high-risk populations, analyses were repeated after excluding: Patients with CCI >8 (n = 12), Patients aged >80 years (n = 15) and IMV recipients (n = 47). Sensitivity analyses confirmed robust ADTP reductions after excluding high-risk subgroups. All comparisons remained statistically significant (Δ = 57–63%, p < 0.01), confirming the intervention’s consistency across subgroups.

**FIGURE 3:**
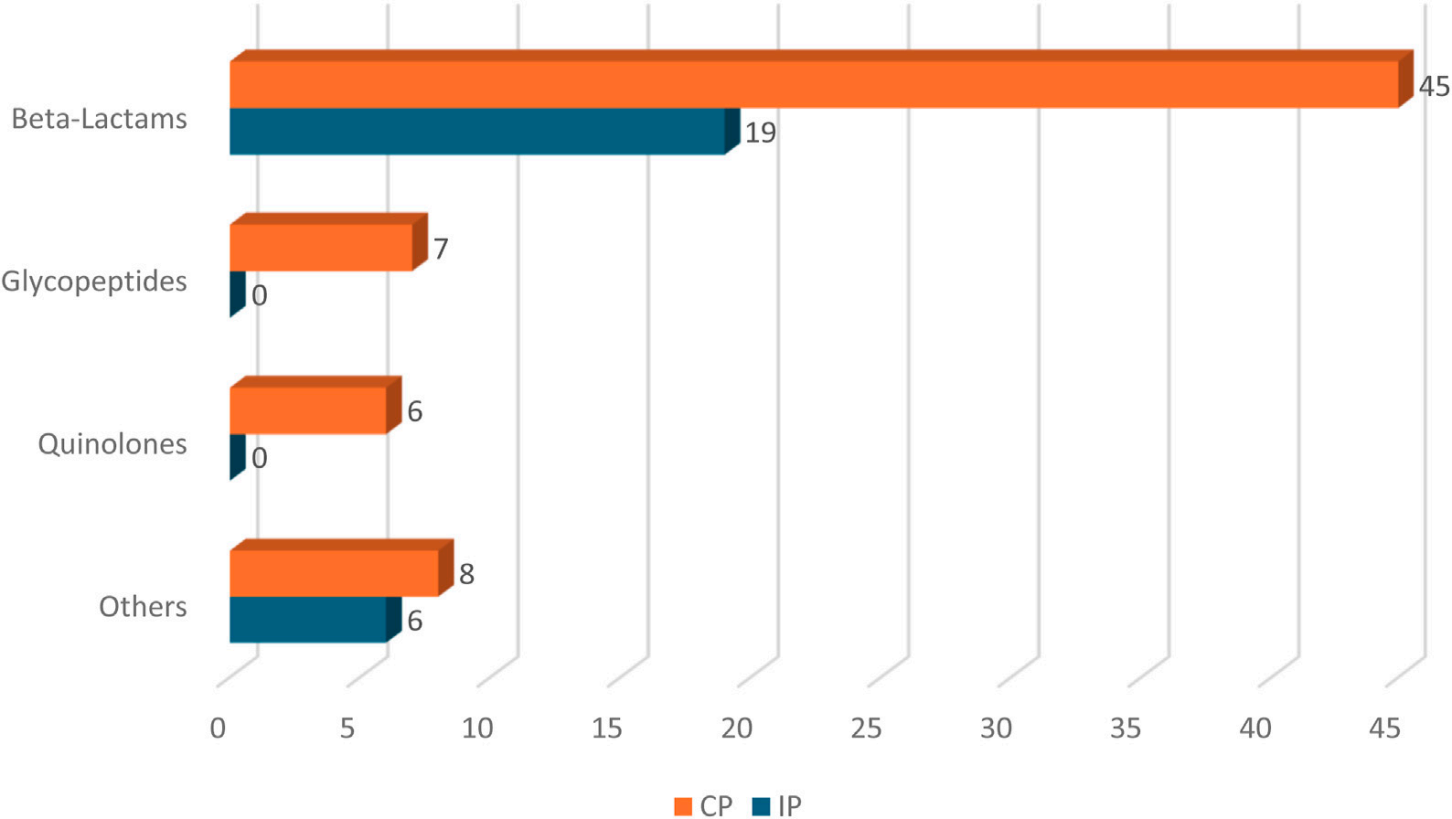
Frequency of Antimicrobial Agents Associated with ADTP CP, Control period; IP, Intervention period.

Figure 3 presents the frequency of antimicrobials associated with ADTPs. In both periods, the drugs most frequently linked to ADTP were from the beta-lactam group (45 *versus* 19). Among the beta-lactams, piperacillin-tazobactam and meropenem were the drugs most frequently associated with ADTP in both periods.

## 4 Discussion

In this prospective, pre-post intervention study conducted in the ICU, ADTPs were identified and categorized over six months—three months of observation followed by 3 months of intervention. During the intervention period, bedside intervention recommendations were provided in response to identified ADTPs. Additionally, regular problem-targeted education sessions were implemented to support antimicrobial therapy optimization. The role of the clinical pharmacist was assessed based on the reduction of ADTPs after clinical pharmacist-led services.

Our ICU predominantly used broad-spectrum agents like piperacillin-tazobactam, meropenem, and vancomycin, consistent with pneumonia leading indication for antimicrobial therapy (56.5%) and guideline-recommended antimicrobials in pneumonia management, all categorized under the Watch group in the WHO AWaRe classification. Vancomycin, that is another Watch group agent, was frequently used for Gram-positive bacterial infections, reflecting its central role in ICU antimicrobial regimens (Evans et al., 2021b; Damas et al., 2015). The low use of Access antibiotics (e.g., ampicillin-sulbactam, cefazolin) is likely attributed to the severity of infections and suspected resistant pathogens in the critically ill population, alongside diagnostic uncertainties in ventilator-associated pneumonia (VAP) management. The diagnostic challenges of VAP, including frequent respiratory tract colonization and non-specific clinical signs, complicate de-escalation efforts and drive the continued use of broad-spectrum antibiotics (Browne et al., 2014; Nussenblatt et al., 2014; Morgan et al., 2017). Given that the second most common reason for starting antimicrobials is sepsis/septic shock (18.8%), Culture-negative sepsis further contributed to difficulties in narrowing antimicrobial therapy, as no identifiable pathogen is found in up to 89% of septic patients in various studies (Gupta et al., 2016; Sigakis et al., 2019; Phua et al., 2013). In our study, pathogens such as *Pseudomonas aeruginosa, Escherichia coli*, and *Klebsiella pneumoniae* were frequently isolated, often necessitating carbapenem use due to their extended-spectrum beta-lactamase production, limiting opportunities for narrower-spectrum antibiotics. *Enterococcus faecium* was another frequently isolated pathogen, often requiring vancomycin therapy.

To effectively address ADTPs, we adopted the terminology and definitions developed by Spivak et al., which offer a more user-friendly and precise approach for antimicrobial therapies compared to alternative classification systems (Spivak et al., 2016). While numerous tools and scales are available in the literature for assessing general drug-related problems, no validated assessment instrument specifically designed for antimicrobial therapy exists to our knowledge (Lim et al., 2018; Basger et al., 2015; Basger et al., 2014). This study represents the first application of Spivak et al.'s terminology in an ICU setting, thus introducing a novel method for the identification and classification of ADTPs in critically ill patients.

A significant portion of the ADTPs identified in our study were categorized as suboptimal, which is concerning in an ICU setting where optimal antimicrobial use is critical. Factors such as increased volume of distribution from fluid resuscitation, renal replacement therapy, and hyperdynamic states may reduce antimicrobial concentrations, particularly for hydrophilic agents like beta-lactams and vancomycin. Furthermore, the presence of pathogens with high minimum inhibitory concentrations (MICs), such as *P. aeruginosa* (the most frequently isolated pathogen in our study), may complicate the achievement of pharmacodynamic targets, even with standard dosing regimens. Given that a substantial portion of ADTPs in our study were categorized as suboptimal, clinical pharmacist-led services were primarily focused on addressing suboptimal ADTPs. The statistically significant reduction in the number of ADTPs observed during the intervention period compared to the observation period (a 62% decrease, particularly regarding sub-optimal category as shown in Table 4) underscores the effectiveness of clinical pharmacist-led services provided in mitigating these problems. In our study, 81.4% of suboptimal therapies were potentially related to inadequate drug exposure, predominantly involving beta-lactams (68.2%) and vancomycin (10.6%). Continuous renal replacement therapy (CRRT) further complicated dosing, often resulting in underdosing due to the altered pharmacokinetics in critically ill patients. Optimizing beta-lactam therapy through prolonged infusions (at least 3 hours) improved target attainment, particularly in septic patients (Arnold et al., 2013; Teo et al., 2014; Dulhunty et al., 2013). Vancomycin, being a hydrophilic agent with a low volume of distribution and predominantly renal excretion, often faces challenges in achieving therapeutic concentrations in critically ill patients (Rybak et al., 2020). The increased volume of distribution and enhanced renal clearance associated with sepsis and augmented renal clearance (ARC) can delay the attainment of target levels, potentially leading to suboptimal vancomycin exposure. These factors necessitate the initiation of treatment with a loading dose to achieve therapeutic targets promptly (Abdul-Aziz et al., 2020).

Inappropriate dose reductions based on estimated glomerular filtration rate (eGFR) during transient AKI were a key ADTP identified in the observation period. Equations like CKD-EPI and Cockcroft-Gault, validated for chronic kidney disease with stable creatinine levels, may be inaccurate in AKI due to fluctuating renal function (Gabardi, 2008; Elston et al., 1993). Ensuring adequate antimicrobial therapy within the first 48 h (standard or near-normal doses) is particularly important in cases of severe infections like sepsis or septic shock. Early dose adjustments based on renal function during this phase may result in subtherapeutic drug levels, potentially leading to poor clinical outcomes, as data showing a 7.6% reduction in survival for each hour of delayed therapy in sepsis. (Kumar et al., 2006; Hassanpour et al., 2021; Mazuski et al., 2016; Crass et al., 2019). Alternative approaches to standard renal clearance equations, such as using urinary creatinine from shorter collection periods (6–8 h), can ensure accurate dosing and avoid toxicity. This method is practical in ICUs where most patients are managed with urinary catheters, offering a cost-effective solution for guiding early dosing decisions. (Cherry et al., 2002; Kadivarian et al., 2022).

Analysis of patient characteristics revealed some imbalances between groups in terms of age, CCI, GCS, and IMV status. The predominance of older patients with high comorbidity (mean CCI = 5.69, estimated mortality = 21%) and higher APACHE II and SOFA scores indicate a challenging population with increased susceptibility to ADTPs, likely due to prolonged and extensive antimicrobial use (Akinosoglou et al., 2023; Yıldız et al., 2020). Additionally, patients on IMV (55.3%) and those with low GCS scores presented higher risks for ADTPs due to the clinical complexities of managing infections like VAP, which often require extended antimicrobial therapy (Semet et al., 2023).

The robustness of ADTPs reduction is underscored by consistent reductions across key subgroups stratified by age, comorbidity burden (CCI), and invasive mechanical ventilation (IMV) status. Notably, patients with higher comorbidity burdens (CCI >5) exhibited the greatest improvement (Δ = 68%), suggesting that pharmacist interventions may disproportionately benefit high-risk populations. Also the weak positive correlation between CCI and ADTPs (r = 0.275, p = 0.011) aligns with the hypothesis that complex patients are more vulnerable to suboptimal antimicrobial use. Sensitivity analyses further validated these findings, as ADTP reductions remained significant even after excluding high-risk subgroups (e.g., CCI >8, age >80 years), reinforcing the intervention’s generalizability.

This study has several limitations. Physician rotation in the ICU team on a monthly basis may have contributed to variability in antimicrobial-related errors, as differences in drug knowledge and the ability to apply pharmacist-led education could have affected clinical decisions. Although no significant drug-drug interactions or specific adverse effects from aggressive antimicrobial therapy in AKI were observed, the complexity of ICU care—particularly in sedated, critically ill, and elderly patients—may have led to undetected adverse drug events. This limits the assessment of antimicrobial safety in this vulnerable population. However, the frequent use of beta-lactams with a wide therapeutic index likely mitigated this risk. The absence of therapeutic drug monitoring (TDM) at the study setting prevented objective confirmation of ADTPs, particularly inadequate dosing, which could have limited influence of our findings on therapy optimization. While the sample size was calculated *a priori* to detect a 22% improvement in dose appropriateness, the final cohort (n = 85) may have limited power to identify smaller effect sizes, increasing the risk of type II errors. Future studies with larger cohorts are warranted to confirm the generalizability of these findings, particularly for less frequent ADTP categories such as unnecessary or inappropriate therapies.

## 5 Conclusion

In conclusion, this study demonstrates that ICUs are places with a high risk of suboptimal antimicrobial use. It emphasizes the need for targeted strategies to optimize the use of antimicrobial agents, especially beta-lactams and vancomycin, through approaches such as use of prolonged infusion and loading doses. Additionally, the findings highlight the vital role of clinical pharmacists in ICUs to lead efforts in optimizing antimicrobial therapy.

### 5.1 Take-home message

This study highlights the high risk of suboptimal antimicrobial use in ICUs and underscores the need for targeted strategies, such as prolonged infusion, loading doses, and use of high-dose of antimicrobials in initial phase of infection treatment, regardless of AKI status, particularly for beta-lactams and vancomycin. The findings further emphasize the critical role of clinical pharmacists in optimizing antimicrobial therapy in these settings.

## Data Availability

The original contributions presented in the study are included in the article/supplementary material; further inquiries can be directed to the corresponding author.

## References

[B1] Abdul-Aziz M. H. Alffenaar J. C. Bassetti M. Bracht H. Dimopoulos G. Marriott D. (2020). Antimicrobial therapeutic drug monitoring in critically ill adult patients: a Position Paper<sub/>. Intensive Care Med. 46 (6), 1127–1153. 10.1007/s00134-020-06050-1 32383061 PMC7223855

[B2] Akinosoglou K. Schoergenhofer C. Rapti V. Gogos C. Dimopoulos G. (2023). The impact of age on intensive care. Ageing Res. Rev. 84, 101832. 10.1016/j.arr.2022.101832 36565961 PMC9769029

[B3] Arnold H. M. Hollands J. M. Skrupky L. P. Smith J. R. Juang P. H. Hampton N. B. (2013). Prolonged infusion antibiotics for suspected gram-negative infections in the ICU: a before-after study. Ann. Pharmacother. 47 (2), 170–180. 10.1345/aph.1R523 23341160

[B4] Basger B. J. Moles R. J. Chen T. F. (2014). Application of drug-related problem (DRP) classification systems: a review of the literature. Eur. J. Clin. Pharmacol. 70 (7), 799–815. 10.1007/s00228-014-1686-x 24789053

[B5] Basger B. J. Moles R. J. Chen T. F. (2015). Development of an aggregated system for classifying causes of drug-related problems. Ann. Pharmacother. 49 (4), 405–418. 10.1177/1060028014568008 25614526

[B6] Browne E. Hellyer T. P. Baudouin S. V. Conway Morris A. Linnett V. McAuley D. F. (2014). A national survey of the diagnosis and management of suspected ventilator-associated pneumonia. BMJ Open Respir. Res. 1 (1), e000066. 10.1136/bmjresp-2014-000066 PMC4275666 25553248

[B7] Cherry R. A. Eachempati S. R. Hydo L. Barie P. S. (2002). Accuracy of short-duration creatinine clearance determinations in predicting 24-hour creatinine clearance in critically ill and injured patients. J. Trauma. 53 (2), 267–271. 10.1097/00005373-200208000-00013 12169932

[B8] Crass R. L. Rodvold K. A. Mueller B. A. Pai M. P. (2019). Renal dosing of antibiotics: are we jumping the gun? Clin. Infect. Dis. 68 (9), 1596–1602. 10.1093/cid/ciy790 30219824

[B9] Damas M. Frippiat F. Ancion A. Canivet J. L. Meuris C. Layios N. (2015). Use of beta-lactams antibiotics in ICU patients. Intensive Care Med. Exp. 3 (Suppl. 1), A1–A1021. 10.1186/2197-425X-3-S1-A1 27419821 PMC4796554

[B10] Dulhunty J. M. Roberts J. A. Davis J. S. Webb S. A. R. Bellomo R. Gomersall C. (2013). Continuous infusion of beta-lactam antibiotics in severe sepsis: a multicenter double-blind, randomized controlled trial. Clin. Infect. Dis. 56 (2), 236–244. 10.1093/cid/cis856 23074313

[B11] Elston A. C. Bayliss M. K. Park G. R. (1993). Effect of renal failure on drug metabolism by the liver. Br. J. Anaesth. 71 (2), 282–290. 10.1093/bja/71.2.282 8123408

[B12] Erb C. T. Metersky M. L. Klompas M. Muscedere J. Sweeney D. A. Palmer L. B. (2016). Management of Adults with hospital-acquired and ventilator-associated pneumonia. Ann. Am. Thorac. Soc. 13 (12), 2258–2260. 10.1513/AnnalsATS.201608-641CME 27925784

[B13] European Centre for Disease Prevention and Control (2019). Healthcare-associated infections acquired in intensive care units. Stockholm, Sweden: ECDC.

[B14] Evans L. Rhodes A. Alhazzani W. Antonelli M. Coopersmith C. M. French C. (2021a). Surviving sepsis campaign: international guidelines for management of sepsis and septic shock 2021. Crit. Care Med. 49 (11), e1063–e1143. 10.1097/CCM.0000000000005337 34605781

[B15] Evans L. Rhodes A. Alhazzani W. Antonelli M. Coopersmith C. M. French C. (2021b). Surviving sepsis campaign: international guidelines for management of sepsis and septic shock 2021. Intensive Care Med. 47 (11), 1181–1247. 10.1007/s00134-021-06506-y 34599691 PMC8486643

[B16] Gabardi S. (2008). Drug dosing in acute kidney injury versus chronic renal insufficiency. Wiley Online Library. 10.1002/9780470987443.ch13

[B17] Gilbert D. N. Chambers H. F. Saag M. S. Pavia A. T. Boucher H. W. Black D. (2023). The Sanford Guide to antimicrobial therapy. Sperryville, VA: Antimicrobial therapy Inc.

[B18] Guclu E. Ogutlu A. Karabay O. Utku A. C. Çag Y. Karahan Z. C. (2017). Antibiotic consumption in Turkish hospitals; a multi-centre point prevalence study. J. Chemother. 29 (1), 19–24. 10.1080/1120009X.2016.1156893 27238248

[B19] Gupta S. Sakhuja A. Kumar G. McGrath E. Nanchal R. S. Kashani K. B. (2016). Culture-negative severe sepsis: nationwide trends and outcomes. Chest 150 (6), 1251–1259. 10.1016/j.chest.2016.08.1460 27615024

[B20] Hassanpour R. Roberts J. A. Lipman J. Udy A. A. Miri M. Ahmadi Koomleh A. (2021). Evaluation of pharmacokinetic and pharmacodynamic parameters of meropenem in critically ill patients with acute kidney disease. Eur. J. Clin. Pharmacol. 77 (6), 831–840. 10.1007/s00228-020-03062-0 33409684 PMC7787627

[B21] Heffernan A. J. Denny K. J. Dulhunty J. M. Cotta M. O. Roberts J. A. Lipman J. (2021). A personalised approach to antibiotic pharmacokinetics and pharmacodynamics in critically ill patients. Anaesth. Crit. Care Pain Med. 40 (6), 100970. 10.1016/j.accpm.2021.100970 34728411

[B22] IBM Watson Health (2023). Micromedex® drug information. Available online at: https://www.micromedexsolutions.com.

[B23] Kadivarian S. Safari S. Sharifi M. Torkamandi H. Alahyari S. Sadeghi M. (2022). Measured versus estimated creatinine clearance in critically ill patients with acute kidney injury: an observational study. Acute Crit. Care. 37 (2), 185–192. 10.4266/acc.2021.01256 35545239 PMC9184982

[B24] Kumar A. Ellis P. Arabi Y. Roberts D. Light B. Parrillo J. E. (2006). Duration of hypotension before initiation of effective antimicrobial therapy is the critical determinant of survival in human septic shock. Crit. Care Med. 34 (6), 1589–1596. 10.1097/01.CCM.0000217961.75225.E9 16625125

[B25] Kumar A. Roberts D. Wood K. E. Light B. Parrillo J. E. Sharma S. (2009). Initiation of inappropriate antimicrobial therapy results in a fivefold reduction of survival in human septic shock. Chest 136 (5), 1237–1248. 10.1378/chest.09-0087 19696123

[B26] Leape L. L. Cullen D. J. Clapp M. D. Burdick E. Demonaco H. J. Erickson J. I. (1999). Pharmacist participation on physician rounds and adverse drug events in the intensive care unit. JAMA 282 (3), 267–270. 10.1001/jama.282.3.267 10422996

[B27] Lim X. Y. Moles R. J. Chaar B. B. Yap K. Z. Chuang S. Li S. C. (2018). Validation of a drug-related problem classification system for the intermediate and long-term care setting in Singapore. Pharmacy 6 (4), 109. 10.3390/pharmacy6040109 30282930 PMC6306714

[B28] Luyt C. E. Bréchot N. Trouillet J. L. Chastre J. (2014). Antibiotic stewardship in the intensive care unit. Crit. Care. 18 (5), 480. 10.1186/s13054-014-0480-6 25405992 PMC4281952

[B29] Marquet K. Liesenborgs A. Bergs J. Vleugels A. Claes N. (2015). Incidence and outcome of inappropriate in-hospital empiric antibiotics for severe infection: a systematic review and meta-analysis. Crit. Care. 19 (1), 63. 10.1186/s13054-015-0795-y 25888181 PMC4358713

[B30] Mazuski J. E. Gasink L. B. Armstrong J. Broadhurst H. Stone G. G. Rank D. (2016). Efficacy and safety of ceftazidime-avibactam plus metronidazole versus meropenem in the treatment of complicated intra-abdominal infection: results from a randomized, controlled, double-blind, phase 3 program. Clin. Infect. Dis. 62 (11), 1380–1389. 10.1093/cid/ciw133 26962078 PMC4872289

[B31] McKenzie C. Spriet I. Hunfeld N. (2024). Ten reasons for the presence of pharmacy professionals in the intensive care unit. Intensive Care Med. 50 (1), 147–149. 10.1007/s00134-023-07285-4 38172297

[B32] Montazeri M. Cook D. J. (1994). Impact of a clinical pharmacist in a multidisciplinary intensive care unit. Crit. Care Med. 22 (6), 1044–1048. 10.1097/00003246-199406000-00027 8205814

[B33] Morgan D. J. Malani P. Diekema D. J. (2017). Diagnostic stewardship—leveraging the laboratory to improve antimicrobial use. JAMA 318 (7), 607–608. 10.1001/jama.2017.8531 28759678

[B34] Nussenblatt V. Avdic E. Berenholtz S. Daugherty E. Hadhazy E. Lipsett P. A. (2014). Ventilator-associated pneumonia: overdiagnosis and treatment are common in medical and surgical intensive care units. Infect. Control Hosp. Epidemiol. 35 (3), 278–284. 10.1086/675279 24521594

[B35] O’Neill J. (2016). Tackling drug-resistant infections globally: final report and recommendations.

[B36] Özlü T. Bübül Y. Alataş F. Arseven O. Çilli A. Çöpü L. (2009). Turkish thoracic society community-acquired pneumonia guideline. Turk. Toraks Deg. 10 (Suppl. 3). 10.5578/tt.70012

[B37] Phua J. Ngerng W. J. See K. C. Tay C. K. Kiong T. Lim H. F. (2013). Characteristics and outcomes of culture-negative versus culture-positive severe sepsis. Crit. Care. 17 (5), R202. 10.1186/cc12896 24028771 PMC4057416

[B38] Pons M. J. Ruiz J. (2019). Current trends in epidemiology and antimicrobial resistance in intensive care units. J. Emerg. Crit. Care Med. 3, 5. 10.21037/jeccm.2019.01.05

[B39] Rybak M. J. Le J. Lodise T. P. Levine D. P. Bradley J. S. Liu C. (2020). Therapeutic monitoring of vancomycin for serious methicillin-resistant *Staphylococcus aureus* infections: a revised consensus guideline. Am. J. Health Syst. Pharm. 77 (11), 835–864. 10.1093/ajhp/zxaa036 32191793

[B40] Schuts E. C. Hulscher M. E. Mouton J. W. Verduin C. M. Stuart J. W. Overdiek H. W. (2016). Current evidence on hospital antimicrobial stewardship objectives: a systematic review and meta-analysis. Lancet Infect. Dis. 16 (7), 847–856. 10.1016/S1473-3099(16)00065-7 26947617

[B41] Semet C. Wallet F. Nseir S. (2023). The ongoing challenge of ventilator-associated pneumonia: epidemiology, prevention, and risk factors for mortality in a secondary care hospital intensive care unit. Infect. Prev. Pract. 5 (4), 100320. 10.1016/j.infpip.2023.100320 38028359 PMC10663678

[B42] Sigakis M. J. G. Jewell E. Maile M. D. Cinti S. K. Bateman B. T. Engoren M. (2019). Culture-negative and culture-positive sepsis: a comparison of characteristics and outcomes. Anesth. Analg. 129 (5), 1300–1309. 10.1213/ANE.0000000000004072 30829670 PMC7577261

[B43] Spivak E. S. Cosgrove S. E. Srinivasan A. (2016). Measuring appropriate antimicrobial use: attempts at opening the black box. Clin. Infect. Dis. 63 (12), 1639–1644. 10.1093/cid/ciw658 27682070 PMC6487652

[B44] Stollings J. L. Foss J. J. Ely E. W. Ambrose A. M. Rice T. W. Girard T. D. (2018). Critical care pharmacists and medication management in an ICU recovery center. Ann. Pharmacother. 52 (8), 713–723. 10.1177/1060028018759343 29457491 PMC6039256

[B45] Teo J. Liew Y. Lee W. Kwa A. L. H. (2014). Prolonged infusion versus intermittent boluses of β-lactam antibiotics for treatment of acute infections: a meta-analysis. Int. J. Antimicrob. Agents. 43 (5), 403–411. 10.1016/j.ijantimicag.2014.01.027 24657044

[B46] Thomnoi T. Phodha T. Riewpaiboon A. Thamlikitkul V. Rattanaumpawan P. (2022). Impact of pharmacist-led implementation of a community hospital-based outpatient parenteral antimicrobial therapy on clinical outcomes in Thailand. Antibiotics 11 (6), 760. 10.3390/antibiotics11060760 35740166 PMC9220076

[B47] Wolters Kluwer (2023). UpToDate® drug information. Available online at: https://www.uptodate.com.

[B48] World Health Organization (2019). The 2019 WHO AWaRe classification of antibiotics for evaluation and monitoring of use. Geneva, Switzerland: WHO.

[B49] World Health Organization (2024). Deaths due to AMR estimated to reach 10 million people by 2050. Geneva, Switzerland: Ministry of Health. Available online at: https://www.who.int/indonesia/news/detail/20-08-2024-deaths-due-to-amr-estimated-to-reach-10-million-people-by-2050.

[B50] Yıldız A. Yiğt A. Benli A. R. (2020). The prognostic role of Charlson comorbidity index for critically ill elderly patients. Eur. Res. J. 6 (4), 378–384. 10.18621/eurj.569123

