# Correction: Clinical pharmacist-led problem-specific education as a strategy for addressing suboptimal antimicrobial use in intensive care unit: a prospective pre-post analysis

**DOI:** 10.3389/fphar.2025.1637512

**Published:** 2025-06-10

**Authors:** Enes Emir İlerler, Yunus Emre Ayhan, Erdem Yalçinkaya, Sait Karakurt, Mesut Sancar

**Affiliations:** ^1^ Department of Clinical Pharmacy, Marmara University, Istanbul, Türkiye; ^2^ Department of Clinical Pharmacy, Prof. Dr. Cemil Taşcıoğlu City Hospital, Istanbul, Türkiye; ^3^ Department of Pulmonary and Critical Care Medicine, Marmara University, Istanbul, Türkiye

**Keywords:** antimicrobial drug therapy problems, intensive care unit, clinical pharmacist-led services, suboptimal antimicrobial use, clinical pharmacists, antimicrobial stewardship

**Affiliation 2** (Department of Clinical Pharmacy, Prof. Dr. Cemil Taşcıoğlu City Hospital, Istanbul, Türkiye) was omitted from the paper. This affiliation has now been added for author Yunus Emre Ayhan.

The authors apologize for this error and state that this does not change the scientific conclusions of the article in any way. The original article has been updated.

